# Exploring the anxiety, depression and perceived burden in advanced cancer: A longitudinal view on patients and caregivers

**DOI:** 10.1017/S1478951525101156

**Published:** 2025-12-29

**Authors:** Talita Caroline de Oliveira Valentino, Carlos Eduardo Paiva, Marco Antonio de Oliveira, Natashe Lemos Dekker, Eduardo Bruera, Lívia Costa de Oliveira, Karla Santos da Costa Rosa, Bianca Sakamoto Ribeiro Paiva

**Affiliations:** 1 Oncology Graduate Program, Barretos Cancer Hospital, Barretos, São Paulo, Brazil; 2 Research Group on Palliative Care and Health-Related Quality-of-Life (GPQual) – Barretos Cancer Hospital, Barretos, São Paulo, Brazil; 3 Faculty Ceres (FACERES), São José do Rio Preto, Brazil; 4 Department of Clinical Oncology–Breast and Gynecology Division, Barretos Cancer Hospital, Barretos, São Paulo, Brazil; 5 Department of Anthropology, University of Amsterdam, Amsterdam, Netherlands; 6 Department of Palliative Care, Rehabilitation and Integrative Medicine and Department of General Oncology, The University of Texas MD Anderson Cancer Center, Houston, TX, USA; 7 Palliative Care Unit, INCA, National Cancer Institute, Rio de Janeiro, Brazil

**Keywords:** Advanced cancer, end-of-life, perceived burden, supportive care, family caregivers, psychological distress

## Abstract

**Background:**

Patients with a life-threatening illness and their family caregivers are often affected by biopsychosocial factors that contribute to suffering and burden-sharing and affect quality-of-life.

**Objectives:**

To compare anxiety and depression levels between patients with incurable cancer and caregivers, investigate the association between perceived burdensomeness and psychological outcomes over time, and evaluate factors associated with perceived burden.

**Methods:**

Secondary analysis of a larger prospective, longitudinal study. Patients with incurable cancer and their family caregivers were interviewed every 3 months, from study enrollment to 12 months, to assess psychological factors. Anxiety and depression were measured with Hospital Anxiety and Depression Scale (HADS) and perceived of burden was assessed using distinct questions directed to patients and caregivers about feeling or perceiving caregiving as a burden. For the data analysis, generalized estimating equations were applied to assess the impact of patient and family caregiver related variables on HADS over time, considering anxiety and depression scores as binary variables.

**Results:**

A total of 190 patient-family caregiver dyads were included. Anxiety was more frequent among family caregivers than patients across all follow-up moments. No significant difference was found in mean depression scores. Feeling like a burden to their family (32.6%) was significantly associated with higher anxiety [odds ratio (OR) = 4.45] and depression scores (OR = 2.73). Poor health perception increased the likelihood of anxiety and depression for patients (OR = 11.00; OR = 38.81) and FC (OR = 2.73; OR = 4.30). Family caregivers demonstrated higher psychological distress, with active employment reducing anxiety (OR = 0.54) and depression (OR = 0.43).

**Significance of results:**

The perceived burden experienced by patients with advanced cancer and their family caregivers over time were factors relevant in the disease process. The feeling of being a burden and poor health perception were key factors contributing to psychological distress, underlining the need for specific interventions in palliative care.

## Introduction

A diagnosis of advanced cancer can be a complex condition that can affect not only patients, but also their family caregivers (FC). Both can face significant physical, psychological, and social challenges such as pain, fatigue, anxiety, depression, perceived burden, and social isolation during the course of the disease (Morrison et al. 2017). Although advances in healthcare, especially in oncology, have improved survival rates for many cancer patients, the burden of living with advanced cancer often leads to a trajectory of suffering (Zhang et al. 2023). Caregivers of cancer patients can suffer an even greater level of burden, in several dimensions including emotional, physical, social, and financial aspects (Wang and Feng 2022), compared to those who care for patients with other diseases (Semere et al. 2021). This is due to increased care demands, reduced personal time, and greater involvement in care processes that are considered more complex (Semere et al. 2021), which leads to a decline in physical health and social functioning (Schandl et al. 2022). As a result, both patients and FC often experience significant psychological and emotional stress (Applebaum and Breitbart 2013; Perez-Cruz et al. 2018) characterized by feelings of anxiety, sadness, hopelessness, and emotional fatigue, which can deeply affect their mental health and daily functioning (Sun et al. 2019; Yuen and Wilson 2021).

Previous studies have demonstrated these findings. For example, a study conducted with patients diagnosed with advanced lung cancer showed a higher risk of emotional problems associated with a lower quality of life and greater symptom burden (Morrison et al. 2017). Another study of patients with advanced lung or noncolorectal gastrointestinal cancer and their caregivers identified a higher prevalence of depressive symptoms in patients and anxiety symptoms among caregivers. The findings indicated that anxiety and depressive symptoms were interrelated among dyads facing incurable diseases, suggesting a bidirectional emotional impact (Jacobs et al. 2017). In the context of cancer patients in palliative care, FC experienced a multidimensional burden, with emotional burden being the most prominent dimension (Zhang et al. 2023). Furthermore, in patients with advanced cancer and other advanced and life-threatening illnesses, the feeling of being a burden is associated with physical, psychological/emotional, existential, and social factors (Rodriguez-Prat et al. 2019).

Patients with advanced cancer often experience feelings of uncertainty and existential anguish, in addition to symptoms of anxiety and depression. They may experience various symptoms and functional decline, especially at the end of life (EOL), requiring assistance with activities such as self-care (Perez-Cruz et al. 2018), which can exacerbate psychological distress. On the other hand, FC, who often have a challenging role in managing care and providing emotional support to the patient, are often exposed to prolonged stress, which increases the risk of mental health problems. In addition to all these demands, the caregiver also has to deal with the reorganization of roles and functions within the family, work-related issues, and their own emotional and psychological needs (Applebaum and Breitbart 2013).

In this sense, this shared burden can affect the well-being of both individuals and their quality of life (Gu et al. 2020; Junkins et al. 2020; Cui et al. 2023). Despite the recognized challenges faced by patients and FC, there is still little understanding of the impact of psychological outcomes, such as anxiety, depression, and perceived burden, which contribute to psychological distress and influence both patients and caregivers over time. Longitudinal investigation of these specific psychological outcomes, together with related contextual factors such as caregiving burden, symptom distress, employment status, and access to healthcare, is essential for understanding the elements that may contribute to suffering. This knowledge is fundamental for developing targeted interventions that meet the emotional and practical needs of patients and caregivers, especially in the context of palliative care. Although some studies have explored these aspects, further research is needed to integrate and expand the existing evidence, particularly in the context of long-term palliative care.

We hypothesized that caregivers would experience higher anxiety and depression than patients and that perceived burdensomeness would be associated with greater psychological distress in both groups. This hypothesis is supported by previous studies which have indicated that patients with advanced and incurable cancer experience a high burden of physical and psychological symptoms since diagnosis (Jacobs et al. 2017; Morrison et al. 2017; Sun et al. 2019; Yuen and Wilson 2021), and their FC are exposed to higher risk of emotional distress (Jacobs et al. 2017; Sun et al. 2019; Yuen and Wilson 2021). Anxiety and depression are interrelated among patients and their FCs (Jacobs et al. 2017), and perceived burdensomeness has been identified as a significant contributor to psychological distress (Jacobs et al. 2017; Cui et al. 2023; Zhang et al. 2023). Thus, the aim of this study was to compare anxiety and depression levels between patients with incurable cancer and caregivers, investigate the association between perceived burdensomeness and psychological outcomes over time, and evaluate factors associated with perceived burden.

## Methods

### Study design, setting and participants

This is a secondary analysis of a larger prospective, longitudinal study conducted at a cancer referral center in Brazil, the Barretos Cancer Hospital, between February 2019 and July 2021. Patients and their primary FC were recruited from the Department of Clinical Oncology and the Palliative Care Unit (Valentino et al. 2023).

Eligibility criteria for patients included: age ≥18 years; a diagnosis of incurable advanced cancer identified based on staging an/or clinician assessment documented in the medical record; ongoing systemic palliative treatment and/or individualized palliative care; an estimated life expectancy of 3–12 months; a functional status of ≤3 according to the Eastern Cooperative Oncology Group (ECOG) (Oken et al. 1982); preserved cognitive abilities (lucid, oriented in the time and space, and spontaneous speech); adequate communication skills (ability to understand, read, and write in Brazilian Portuguese, as well as spontaneous speech), assessed by the researcher who approached and interviewed the participants; and the presence of a primary caregiver. FCs were eligible if they were aged ≥18 years, aware of the patient’s diagnosis of incurable advanced cancer, and able to provide a contact phone number for follow-up. Patient-FC dyads were excluded if either member declined to participate in the study (Valentino et al. 2023).

### Assessment instruments and data collection

The present study was approved by the Research Ethics Committee of the Barretos Cancer Hospital under protocol number 2.007.644. Written informed consent was obtained prior to study participation. Structured interviews were conducted both in person and via telephone by a single nurse researcher experienced in the field (TCOV) (Valentino et al. 2023).

Study participants were followed for a 12-month period. At baseline (M0), patients and FC participated in in-person interviews conducted separately to ensure the independence of their responses. Subsequently, telephone interviews were verbally administered with participants at 3-month intervals across four follow-up assessments (M1, M2, M3, M4). The interview questions can be found in supplementary material 1.

Sociodemographic and clinical characteristics of the study participants were collected, e.g., gender, age, marital status, education, professional activity, living place characteristics, health self-perception, caregiver’s relationship with patient, cancer treatment, performance status, symptoms assessed by the validated and culturally adapted Portuguese version of the Edmonton Symptom Assessment Scale (ESAS) (Paiva et al. 2015), originally developed by Bruera et al. (1991), medicine regular use, and others.

To assess psychological factors in both patients and FC, the validated and culturally adapted Portuguese version of the Hospital Anxiety and Depression Scale (HADS) (Botega et al. 1995), originally developed by Zigmond and Snaith (1983). This scale is effective for identifying symptoms of anxiety and depression, is easy to understand, quick to administer, and, in clinical practice, can play a useful role in detecting patients requiring psychological care. The scale consists of 14 items – seven related to anxiety symptoms and seven to depression symptoms – answered on a four-point Likert scale. Scores of 8 or higher were considered positive based on the scale’s validation process for anxiety (HADS ≥ 8) and depression (HADS ≥ 8) (Botega et al. 1995).

Regarding the perceived burden, participants were asked two distinct questions. Patients were asked, “Do you feel like a burden to your family?” (yes vs. no). Caregivers were asked, “Is caring for the patient, your loved one, a burden for you?” (yes vs. no) at each follow-up moment (M0-M4). Both questions were based on an adapted model of Chochinov, considering it as part of the context of preserving the repertoire of dignity (Chochinov et al. 2002), i.e., within the framework of the Social Dignity Inventory, which includes the perception of oneself as a burden (Chochinov et al. 2002). A dichotomous (yes/no) variable was adopted to measure perceived burden, allowing participants to express their experiences in their own terms.

### Statistic

Descriptive statistics (mean, standard deviation, absolute and relative frequency) and plots were used to summarize patients’ and caregivers’ characteristics. Chi-square or Fisher’s exact test and *t*-test were used to examine the difference between the groups (patients versus FC) about perceived burden, anxiety, and depression at each time point separately. We applied generalized estimating equations (GEEs) using HADS (anxiety) and HADS (depression) as binary dependent variable. The models were fitted considering a binomial distribution and LOGIT as the link function. We included the subject identification number as a random effect in the statistical model, with five evaluation time points as a within-subject variable, and an independent correlation matrix was adopted. Three models were adjusted for each dependent variable (six models in total). In Model 1, the variable group (patients/FC), along with patient-related and family caregiver (FC)-related variables, were considered as independent variables. Model 2 was adjusted for patient-related variables, while Model 3 was adjusted for FC-related variables. The final models included only the variables with a *P*-value < 0.05 in the Wald test, selected using the backward method. No potential confounders were used in the model adjustments.

The data were recorded using the Research Electronic Data Capture Platform (REDCap) (Harris et al. 2009). Statistical Package for the Social Sciences (SPSS) software (version 27; SPSS, Inc., Chicago, IL) was used to perform the statistical analysis, and we considered 5% as the significance level.

## Results

### Patient and family caregiver characteristics

A total of 190 patients and 190 FC were included in the study. Forty-two patients completed all stages of the study, while 148 patients and one FC died during the follow-up period.

The mean age (standard deviation) of patients and FC was 59.3 (13.1) and 48.5 (13.8) years, respectively. Among patients, 100 (52.6%) were female, compared to 140 (73.7%) of caregivers. The majority of both patients (*n* = 130; 68.4%) and caregivers (*n* = 132; 69.5%) were married or living as married. Most patients had less than 8 years of formal education (*n* = 130; 68.4%), while 79 (41.6%) caregivers had between 8 and 11 years of education (reference cross-sectional study) (Valentino et al. 2022).

Sixty percent of FC (*n* = 114) reported living with the patient, and 40% (*n* = 76) had interrupted their professional activities to provide care. The predominant relationship between FC and patient was spouse (40.5%; *n* = 77), followed by child (27.9%; *n* = 53) and sibling (9.5%; *n* = 18). At baseline, 47.9% (*n* = 99) of patients were receiving standard oncology treatment combined with palliative care. Regarding performance status, 41.1% (*n* = 78) and 36.3% (*n* = 69) had an ECOG performance status of 3 and 2, respectively. Additionally, 92.1% (*n* = 175) reported experiencing one or more symptoms assessed by the Edmonton Symptom Assessment Scale (ESAS), and 93.2% (*n* = 177) were regularly using one or more medications. Comprehensive sociodemographic and clinical profiles of the participants were detailed in a previous publication (Valentino et al. 2022).

### Anxiety and depression

Table 1 shows that anxiety (HADS ≥ 8) was more frequent among FC in all study follow-up assessments (M0: 40.5%, *n* = 68 vs. 31.5%, *n* = 53; M1: 33.9%, *n* = 41 vs. 13.4%, *n* = 15; M2: 30.1%, *n* = 22 vs. 14.7%, *n* = 10; M3: 25.9%, *n* = 14 vs. 12.5%, *n* = 6; M4: 23.1%, *n* = 9 vs. 18.4%, *n* = 7). Regarding depression (HADS ≥ 8), the frequencies were nearly equivalent between patients and their FC.

**Table 1.** Analysis of the perceived burden, anxiety and depression of patients and family caregivers over time (M0–M4)

Variables		Patients *n* (%)	Family C–caregivers *n* (%)	Mean difference^b^ (95% CI)	*P*
**M0 (baseline)**		**(*n* = 168)**	**(*n* = 168)**		
**Anxiety**	**Yes (HADS ≥ 8)**	53 (31.5)	68 (40.5)		0.089*
	**Mean (SD)**	5.87 (4.63)	7.41 (4.78)	−1.54 (−2.55 to −0.53)	0.003**
**Depression**	**Yes (HADS ≥ 8)**	60 (35.7)	52 (31.0)		0.419*
	**Mean (SD)**	6.18 (4.63)	5.55 (4.17)	0.63 (−0.32 to 1.56)	0.194**
**Burden^a^**	**Yes**	65 (38.5)	1 (0.6)		<0.001*
**M1 (Follow-up 3 months)**		**(*n* = 112)**	**(*n* = 121)**		
**Anxiety**	**Yes (HADS ≥ 8)**	15 (13.4)	41 (33.9)		0.454*
	**Mean (SD)**	4.07 (3.53)	6.54 (4.59)	−2.47 (−3.51 to −1.41)	<0.001**
**Depression**	**Yes (HADS ≥ 8)**	26 (23.2)	34 (28.1)		0.454*
	**Mean (SD)**	4.79 (4.29)	5.21 (4.38)	−0.42 (−1.54 to 0.70)	0.461**
**Burden^a^**	**Yes**	34 (30.4)	0 (0.0)		<0.001*
**M2 (Follow-up 6 months)**		**(*n* = 68)**	**(*n* = 73)**		
**Anxiety**	**Yes (HADS ≥ 8)**	10 (14.7)	22 (30.1)		0.043*
	**Mean (SD)**	3.62 (3.97)	5.19 (3.74)	−1.57 (−2.85 to −0.29)	0.017**
**Depression**	**Yes (HADS ≥ 8)**	12 (17.6)	15 (20.5)		0.676*
	**Mean (SD)**	3.93 (3.60)	4.25 (4.03)	−0.32 (−1.59 to −0.95)	0.621**
**Burden^a^**	**Yes**	21 (30.9)	0 (0.0)		<0.001*
**M3 (Follow-up 9 months)**		**(*n* = 48)**	**(*n* = 54)**		
**Anxiety**	**Yes (HADS ≥ 8)**	6 (12.5)	14 (25.9)		0.133*
	**Mean (SD)**	3.77 (3.10)	5.43 (4.29)	−1.66 (−3.11 to −0.19)	0.027**
**Depression**	**Yes (HADS ≥ 8)**	9 (18.8)	12 (22.2)		0.807*
	**Mean (SD)**	4.10 (4.53)	4.07 (3.70)	0.03 (−1.58 to 1.64)	0.971**
**Burden^a^**	**Yes**	13 (27.1)	0 (0.0)		<0.001*
**M4 (Follow-up 12 months)**		**(*n* = 38)**	**(*n* = 39)**		
**Anxiety**	**Yes (HADS ≥ 8)**	7 (18.4)	9 (23.1)		0.780*
	**Mean (SD)**	3.71 (3.58)	4.64 (3.84)	−0.93 (−2.61 to 0.75)	0.275**
**Depression**	**Yes (HADS ≥ 8)**	8 (21.1)	7 (17.9)		0.780*
	**Mean (SD)**	4.05 (4.92)	3.82 (3.68)	0.23 (−1.73 to 2.20)	0.815**
**Burden^a^**	**Yes**	9 (23.7)	0 (0.0)		<0.001*

Abbreviations: SD, standard deviation; HADS, Hospital Anxiety and Depression Scale; (95%CI), 95% Confidence Interval.

a Feels like a burden to the family/ Patient care is a burden. *Chi-square test; **T-student test; *P*-value < 0.05.

b Difference between the mean anxiety/depression of the patient and the mean anxiety/depression of the family caregiver.

The mean anxiety score of FC was higher than that of patients at all follow-up time points (M0: 7.41 vs. 5.87, *P* = 0.003; M1: 6.54 vs. 4.07, *P* < 0.001; M2: 5.19 vs. 3.62, *P* = 0.017; M3: 5.43 vs. 3.77, *P* = 0.027; M4: 4.64 vs. 3.71, *P* = 0.275). No statistically significant difference was observed in the mean depression scores (Table 1).

The analysis of the interaction between group and follow-up time points on psychological outcomes demonstrated that for the anxiety model, there was no significant change in the trend between the groups over time (*P =* 0.133). On the other hand, for the depression model, there was a significant change in the trend between the groups over time (*P =* 0.039). At baseline (M0), the patient group had a lower likelihood of depression when compared to caregivers (OR = 0.98; CI95% 0.31–3.14). At subsequent moments, patients showed a higher likelihood of depression when compared to caregivers (M1, OR = 2.67; M2, OR = 1.47; M3, OR = 1.51; M4, OR = 1.00).

In the patient group, anxiety changed significantly over time between M0 – M1 (*P <* 0.001), M0 – M2 (*P =* 0.036), and M0 – M3 (*P =* 0.019). In the caregiver group, anxiety also changed significantly between the same moments (*P <* 0.001). Regarding depression, significant changes were observed in the patient group between M0–M1 (*P <* 0.001) and M0–M2 (*P =* 0.032). In the caregiver group, depression also changed significantly between M0 and M1 (*P =* 0.016).

### Perceived burden

A total of 142 patients (32.6%) reported feeling like a burden to their FC. Only one FC, at M0, reported that care for the patient was a burden (Table 1).

**Figure 1.**
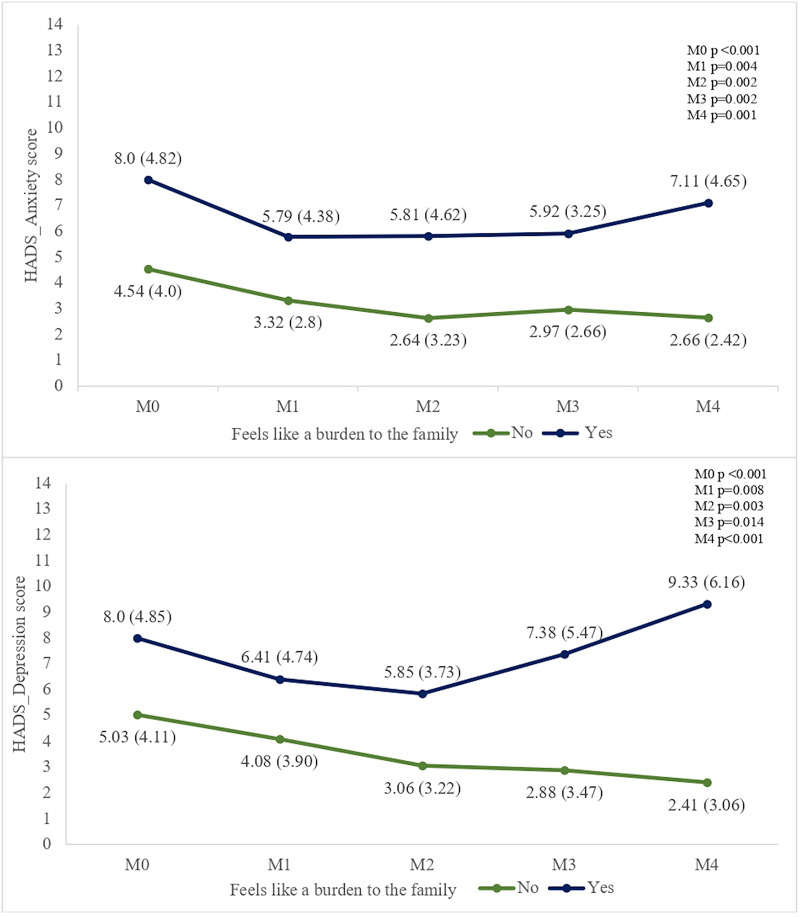
Association between perceived burden and the mean scores (standard deviation) of anxiety and depression of patients over time (M0–M4).

In the analysis of the association between perceived burden and the mean anxiety and depression scores of patients over time (M0–M4), the group of patients who felt like a burden to their family was significantly associated with higher mean anxiety (M0: 8.0; M1: 5.79; M2: 5.81; M3: 5.92; M4: 7.11) and depression scores (M0: 8.0; M1: 6.41; M2: 5.85; M3: 7.38; M4: 9.33) at all follow-up time points when compared to the group of patients who did not feel like a burden to their family (Figure 1).

At M0 (baseline) and M4 (12-month follow-up), the highest anxiety (M0: 8.0 vs. 4.54, *P <* 0.001; M4: 7.11 vs. 2.66, *P =* 0.001) and depression scores (M0: 8.0 vs. 5.03, *P <* 0.001; M4: 9.33 vs. 2.41, *P <* 0.001) were observed between the groups. A trend of decreasing mean anxiety and depression scores was noted among patients who did not feel like a burden compared to those who did feel like a burden to their family (Figure 1). In addition, feeling like a burden to their family was significantly associated with poor self-perception of health at M3 (*P <* 0.001) and M4 (*P =* 0.001) compared to patients who did not feel like a burden to their family.

**Figure 2.**
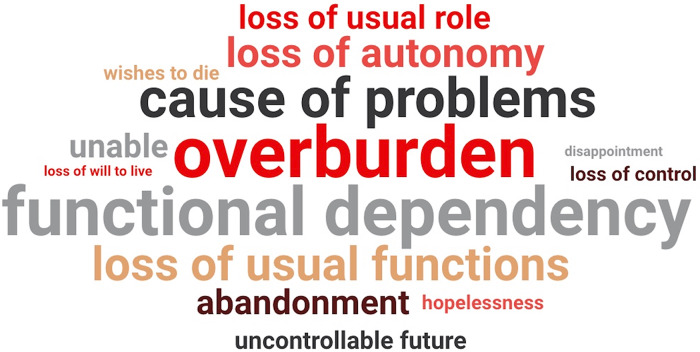
Word clouds generated using patients’ narratives regarding the psychosocial burden.

At M0 (baseline) and M4 (12-month follow-up), the highest anxiety (M0: 8.0 vs. 4.54, p<0.001; M4: 7.11 vs. 2.66, p=0.001) and depression scores (M0: 8.0 vs. 5.03, p<0.001; M4: 9.33 vs. 2.41, p<0.001) were observed between the groups. A trend of decreasing mean anxiety and depression scores was noted among patients who did not feel like a burden compared to those who did feel like a burden to their family (Figure 1). In addition, feeling like a burden to their family was significantly associated with poor self-perception of health at M3 (p<0.001) and M4 (p=0.001) compared to patients who did not feel like a burden to their family. According to the participants’ narratives regarding the psychosocial burden, a synthesis of patients´ responses was made in two or more relevant words and word clouds were generated, thus summarizing the findings. The words ‘‘overburden’’ (n=96; 35.68%), ‘‘functional dependency’’ (n=73; 27.13%), “cause of problems” (n=31; 11.52%), “loss of usual functions” (n=18; 6.69%) and “loss of autonomy” (n=12; 4.46%) were more frequent (Figure 2).

### Factors associated with anxiety and depression

The univariate analysis identified variables that were significantly associated at some moment during follow-up with anxiety and depression (HADS ≥ 8 vs. HADS < 8). The multivariate model, adjusted to include group (patient/FC), professional activity, and gender as independent factors, estimated that FC were more likely to experience anxiety (OR = 2.93; *P <* 0.001) and depression (OR = 1.79; *P =* 0.041). Active employment was associated with a reduced likelihood of anxiety (OR = 0.54; *P =* 0.023) and depression (OR = 0.43; *P =* 0.004). Male gender was significantly associated with a lower likelihood of depression (OR = 0.39; *P <* 0.001) (Table 2).

**Table 2.** Multivariate analysis of factors associated with anxiety and depression over time (Model 1)

	Anxiety (HADS ≥ 8)			Depression (HADS ≥ 8)		
Variables	Category	OR (CI 95%)	*P*	Category	OR (CI 95%)	*P*
Groups	Patients	1	–	Patients	1	–
	Family caregivers	2.93 (1.74–4.94)	*<0.001*	Family caregivers	1.79 (1.02–3.15)	*0.041*
Professional activity	Inactive	1	–	Inactive	1	–
	Active	0.54 (0.32–0.91)	*0.023*	Active	0.43 (0.24–0.76)	*0.004*
Gender	–	–	–	Female	1	–
	–	–	–	Male	0.39 (0.25–0.61)	*<0.001*

Abbreviations: HADS, Hospital Anxiety and Depression Scale; OR (CI 95%), odds ratio (confidence interval 95%). Multivariate logistic regression analysis. *P*-value < 0.05 Wald test.

Table 3 presents the individual factors for patients and FC related to anxiety and depression based on the adjusted multivariate regression model. Among patients, feeling like a burden to their family increased the likelihood of anxiety (OR = 4.45; *P =* 0.003), male patients reported a lower depression score than female patients (OR = 0.18; *P <* 0.001). Poor self-perception of health significantly increased the likelihood of anxiety and depression for both patients (OR = 11.00, *P <* 0.001; OR = 38.81, *P <* 0.001) and FC (OR = 2.73, *P =* 0.006; OR = 4.30, *P =* 0.006).

**Table 3.** Multivariate analysis of factors associated with anxiety and depression on the patients and family caregivers over time

	Anxiety Yes (HADS ≥ 8)			Depression Yes (HADS ≥ 8)		
Variables	Category	OR (CI 95%)	*P*	Category	OR (CI 95%)	*P*
**Patients’ model (Model 2)**						
Health self-perception	Good	1	–	Good	1	–
	Regular	4.45 (1.68–11.79)	*0.003*	Regular	3.31 (1.47–7.44)	*0.004*
	Poor	11.00 (3.50–34.59)	*<0.001*	Poor	*38.81 (8.38–179.58)*	*<0.001*
Feels like a burden to the family	No	1	–	–	–	–
	Yes	4,45 (1,68–11,79)	*0.003*	–	–	–
Gender	–	–	–	Female	1	–
	–	–	–	Male	0.18 (0.77–0.45)	*<0.001*
**Family caregivers’ model (Model 3)**						
Health self-perception	Good	1	–	Good	1	–
	Regular	2.73 (1.41–5.29)	*0.003*	Regular	2.89 (1.39–6.02)	*0.004*
	Poor	2.73 (1.41–5.29)	*0.006*	Poor	4.30 (1.53–12.12)	*0.006*
Place for accommodations and patient care	No	1	–	–	–	–
	Yes	2.60 (1.21–5.58)	*0.014*	–	–	–

Abbreviations: HADS, Hospital Anxiety and Depression Scale; OR (CI 95%), odds ratio (confidence interval 95%). Multivariate logistic regression analysis. *P*-value < 0.05 Wald test.

Conversely, FC who had a designated space in their home for accommodating and caring for the patient, their loved one were significantly associated with an increased likelihood of anxiety (OR = 2.60; *P =* 0.014) (Table 3). The estimates reflect the effect of the covariates over the entire follow-up period, rather than at a specific time point.

## Discussion

This study presents important results regarding the perceived burden of patients with advanced cancer and their FC. First, we observed a greater burden of anxiety in FC than in patients. Second, it was possible to identify that the feeling of being a burden and the perception of poor health were factors associated with a greater likelihood of psychological distress in patients with advanced cancer and FC. Knowledge of this evidence may support better planning of patient- and family-centered care, involving addressing these and other important multidimensional aspects in this context.

Psychosocial suffering is a prevalent and clinically important complication that occurs throughout the course of the oncology disease and can be exacerbated in the more advanced stages (Riba et al. 2023). Thus, to plan care centered on the patient-family binomial, it is necessary to understand that, in parallel with the progression of cancer, it is common to see an increase in the burden of symptoms and the occurrence of a progressive functional decline that reflects in an increase in suffering and the demand for support from FC. In addition to these factors, the presence of symptoms such as mental confusion, behavior change, cachexia, and uncontrolled pain are related to the burden of psychosocial suffering in FC (Hasdenteufel and Quintard 2022). Compared to FC of patients diagnosed with other life-threatening illnesses, FC of cancer patients face an even greater burden, resulting in a reduction in physical condition and social function (Schandl et al. 2022).

FC, in turn, suffer from a high emotional impact generated by multiple factors, including difficulties related to the need to adapt quickly to clinical/functional decline in order to maintain effective patient care and the fear of dealing with death and the imminent loss of their loved one (Palacio et al. 2018). In addition, there are multiple other responsibilities of the FC, including the need to make important decisions, carrying out their own activities of daily living and self-care, which can end up being put on the back burner (Penner et al. 2012). The disease has an impact on the lives of patients and their families, especially the FC.

In this context, FC can face a variety of challenges caused by the prolonged burden of caring for advanced cancer patients (Rassouli et al. 2023). In contrast to depression, we found that the frequency and average scores of anxiety were higher in caregivers than in patients at all assessment times. A previous study assessed the FC burden and psychological well-being of FC patients with hematological and nonhematological cancer. FC burden was found to be positively associated with symptoms of depression and anxiety. However, as perceived social connectedness increased, the relationship between FC burden and depression decreased; on the other hand, this relationship was not observed for anxiety (Yuen and Wilson 2021). Importantly, the levels of distress reported by cancer FC are often higher than those reported by FC of patients with chronic nonmalignant diseases, highlighting the unique challenges of caring for cancer patients (Sun et al. 2019; Yuen and Wilson 2021). This fact points to the need for the health team to pay attention to these caregivers and makes it possible to reflect on care for the caregiver (Dias et al. 2018). To ensure that FC are able to care for patients with advanced cancer, there is a need to detect and adequately address any sources of distress that may plague them (Palacio et al. 2018).

In addition, we found that patients who felt like a burden to their family had higher levels of anxiety and depression throughout follow-up. This feeling of being a burden is frequent in patients with advanced diseases and is a clinically important issue due to the suffering, reduced quality of life, and sense of dignity it can cause (Rodriguez-Prat et al. 2019). This feeling may also affect the patient’s decision-making about their therapeutic possibilities (Monforte-Royo et al. 2011).

Aspects from different dimensions can explain the origin of this feeling of being a burden. Feelings of burden are associated with physical, psychological/emotional, existential, and social factors and cannot be understood without taking into account the patients’ personal interpretation of their dependency or care needs, and, therefore, it is also necessary to understand their biographical background. It usually emerges because patients feel frustrated and guilty due to the difficulties and excessive demands they place on their FC (Rodriguez-Prat et al. 2019).

Qualitative studies highlight the burdens caregivers face in their daily lives, including restricted freedom, social isolation, and limited time for leisure. However, during interviews, the caregivers openly discuss these challenges, which can be considered a positive aspect, as it facilitates a clearer understanding of their unmet needs and potential strategies to address them (Geerlings et al. 2023; Sherman et al. 2014). Notably, only one caregiver explicitly reported feeling that caring for their care partner was a burden. This is a striking finding, suggesting that while caregivers face significant challenges, they may not necessarily perceive their role as burdensome, or they may be reluctant to express such feelings.

The results of the multivariate analysis pointed to the fact that the feeling of being a burden and the perception of poor health were factors associated with a greater likelihood of psychological distress in patients with advanced cancer and FC. Zhang et al. (2023) describe the multiple dimensions that are part of the burden of the FC of patients with advanced cancer in the palliative setting, including the physical dimension (many care tasks and poor health condition), emotional (strong negative emotions resulting from the suffering of patients and insufficient and ineffective family communication), social (less social interaction and conflict of social function) and economic burdens, factors that aggravate the burden (prevention and control of COVID-19 and marital relationship with patients) and factors that mitigate the burden (social support). All these dimensions may contribute to understanding the greater propensity to anxiety (OR = 2.93; *P <* 0.001) and depression (OR = 1.79; *P =* 0.041) among the FC in this study.

Among the patients, feeling like a burden to the family increased the likelihood of anxiety (OR = 4.45; *P =* 0.003), as did low self-perception of health significantly increasing the likelihood of anxiety and depression (OR = 11.00, *P <* 0.001; OR = 38.81, *P <* 0.001). The feeling of being a burden should be understood as a complex issue that can reflect a patient’s distress in different spheres. According to a systematic review study by Rodriguez-Prat et al. (2019), in the majority of the studies included, patients explained how the consequences of the disease process (physical deterioration, loss of function, incontinence, cognitive impairment, among others) affected their sense of identity and, through this perception, they saw themselves as a burden to others.

Regarding low self-perception of health, there was a significant increase in the likelihood of anxiety and depression in FC (OR = 2.73, *P =* 0.006; OR = 4.30, *P =* 0.006). The self-perceived health of FC has been pointed out as one of the risk factors related to the ability of this FC to provide the necessary care to the patient (Valente et al. 2011), as well as the report of good or excellent self-care was associated with lower caregiver burden (OR = 0.39, 0.21–0.73 CI, *P =* 0.003) [36]. On the other hand, our findings observed that FC who had a designated space in their home to accommodate and care for the patient, their loved one, were significantly associated with a greater likelihood of anxiety (OR = 2.60; *P =* 0.014). As well as being involved in direct patient care, FC may also have other responsibilities, such as caring for other dependents, household chores, and financial worries (Ulgen and Ugur 2022; Teo et al. 2023). In some cultures, children taking responsibility for the care of their sick parents is a valued quality, although it can increase feelings of social pressure (Borges et al. 2017; Teo et al. 2023).

Regarding the interaction between the groups (patients and caregivers) and the follow-up moments in the psychological outcomes, they showed that over time, patients with terminal illnesses can be affected by psychological distress, such as depression. In addition, the psychological outcomes of anxiety and depression may differ between the groups during the course of the disease. The findings corroborate with the literature, and are not limited only to the patient, but also to their FC who accompany their loved one through this process (Lo et al. 2010; Sewtz et al. 2021; Seiler et al. 2024). Due to the existing relationship and the degree of interdependence, the family caregiver can suffer equally and have their quality-of-life and health affected over time (Seiler et al. 2024). Thus, support strategies can be implemented to mitigate this psychological suffering throughout the evolutive process of the disease, such as care plans and interventions that prioritize the promotion of dignity (Monforte-Royo et al. 2018; Seiler et al. 2024).

This study has some limitations. First, the study was carried out in a single cancer center, which may limit the generalizability of the results to other services. However, we believe that the cultural and regional effects of the Brazilian population can be represented by the institution in the study, as it treats patients from all five regions of the country. Second, assessments of perceived burden over time can be influenced by external factors, such as social support, access to health services, changes in oncological treatment and support, progression of the disease, or deterioration in the health of caregivers. To minimize this impact, this and other information from both patients and their caregivers was assessed over time. Third, this study assessed perceived burden using a dichotomous (yes/no) variable that allowed patients and FC to express their experiences in their own terms in relation to perceiving oneself as a burden. While this approach is in keeping with the qualitative and phenomenological nature of dignity research, it does not provide a psychometric assessment. Fourth, it’s a prospective cohort study with a population of patients with advanced cancer, estimated survival ≤ 12 months, and their FC. The loss of follow-up due to the death of patients over time reduces the size of the sample and influences the statistical analysis. However, the results of this study made it possible to plan new research on this subject, which is still limited, especially in Brazil, and could have significant implications for public policies and the healthcare needed to support patients and their families. Further research is needed, with assessments over time, to better understand the factors associated with perceived burden among patients and caregivers from different cultures during the course of the disease and at the end-of-life.

## Conclusion

This study highlights the significant perceived burden, anxiety, and depression experienced by both patients with advanced cancer and their FC, with caregivers in particular reporting higher levels of anxiety. Key factors, such as the perception of being a burden and poor health perception, were found to significantly contribute to psychological distress. These findings underscore the critical need for targeted palliative care interventions that address both the emotional and practical needs of patients and FC. This includes providing psychological support, exploring solutions like employment assistance for FC, and developing personalized care models that more effectively meet the unique needs of both groups.

## Supporting information

10.1017/S1478951525101156.sm001 Valentino et al. supplementary material 1 Valentino et al. supplementary material

10.1017/S1478951525101156.sm002 Valentino et al. supplementary material 2 Valentino et al. supplementary material

## Data Availability

Data supporting the findings of this study are available from the corresponding author upon reasonable request.

## Supplementary material

The supplementary material for this article can be found at https://doi.org10.1017/S1478951525101156/.
