# Supplementary figures and images for: Exploring the anxiety, depression and perceived burden in advanced cancer: A longitudinal view on patients and caregivers
Source: Palliat Support Care. 2025 Dec 29;24:e17. doi: 10.1017/S1478951525101156 (PMC13166427; doi:10.1017/S1478951525101156)

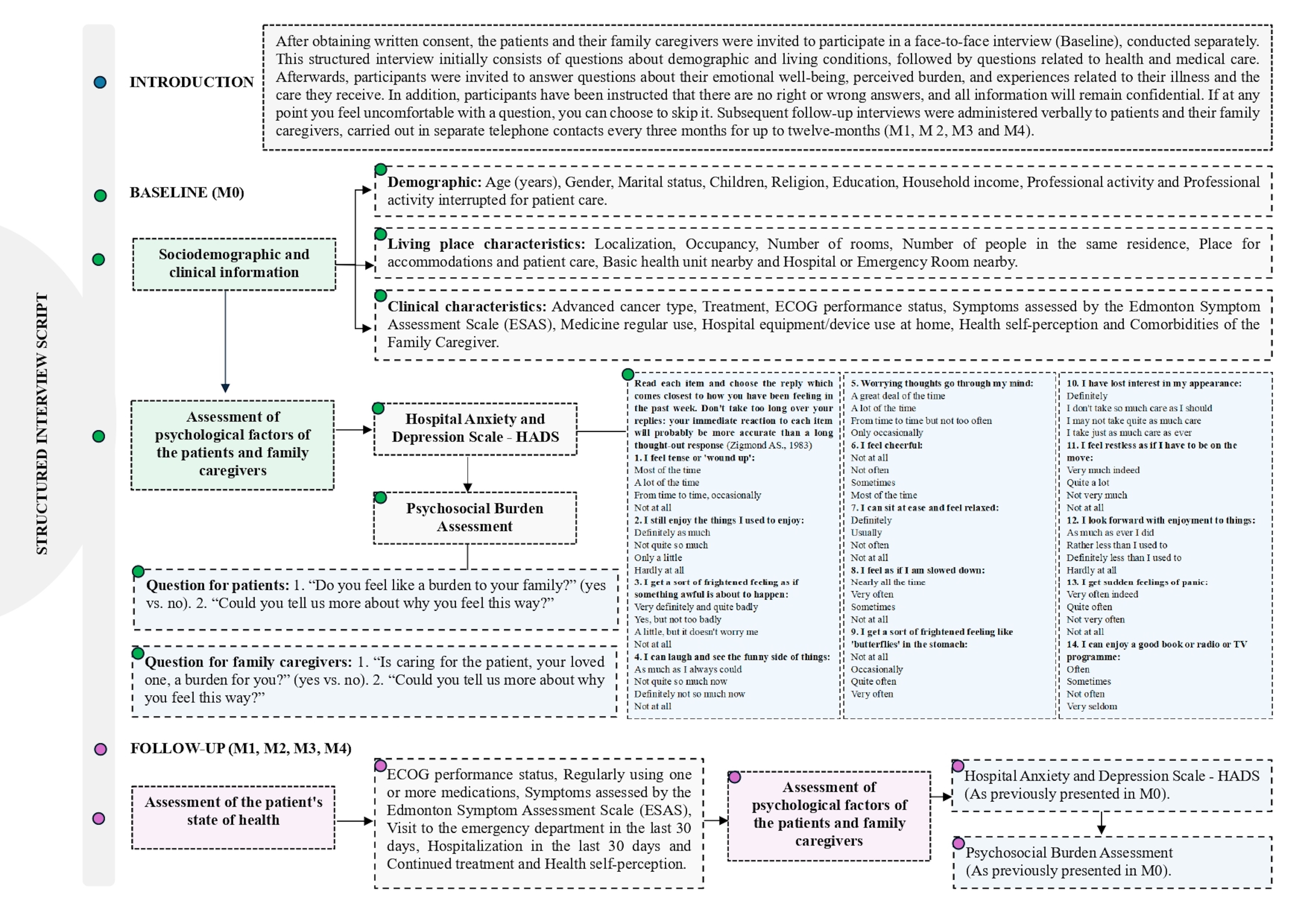

Supplement: Valentino et al. supplementary material 1 — Valentino et al. supplementary material [file S1478951525101156sup001.jpg]

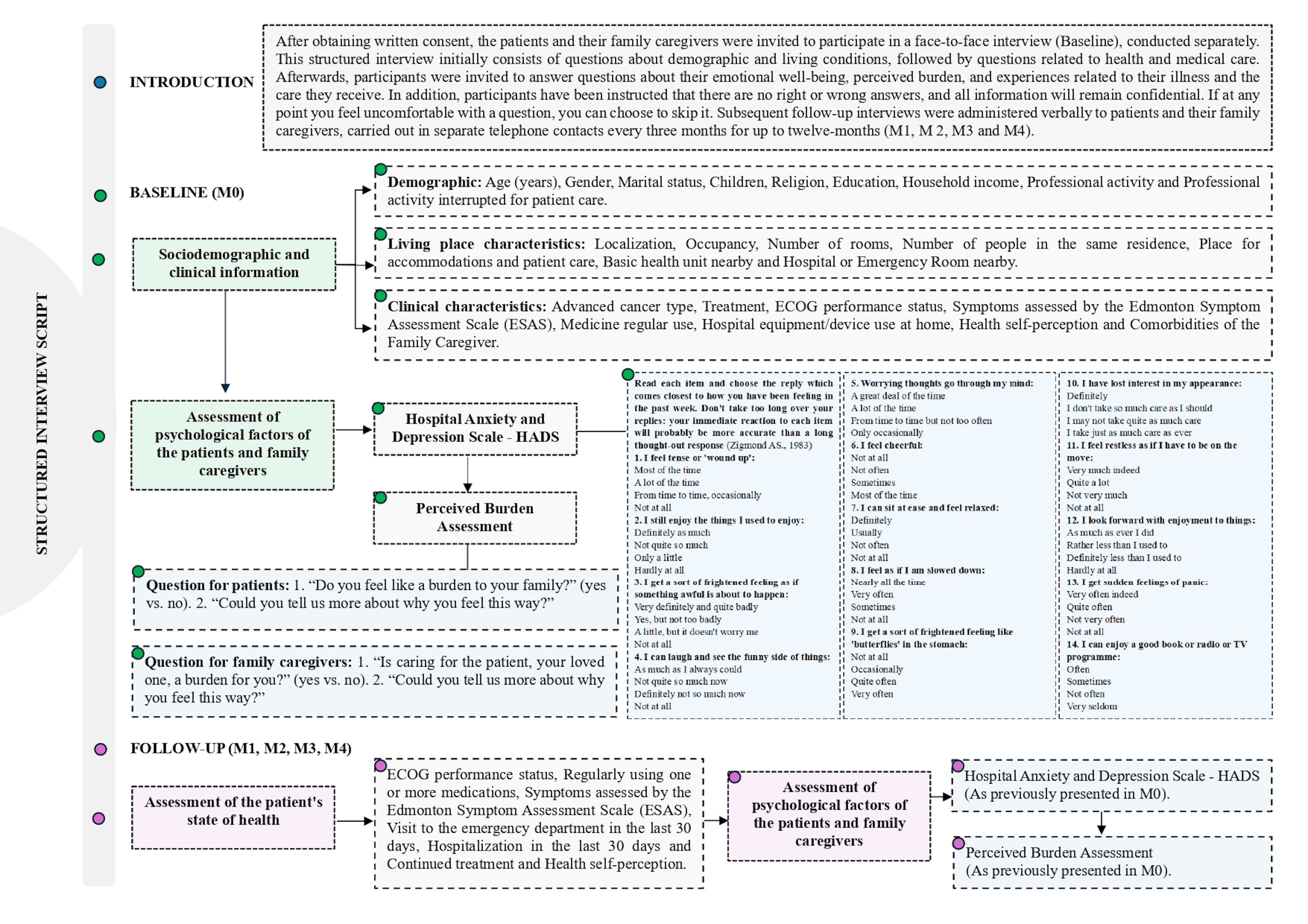

Supplement: Valentino et al. supplementary material 2 — Valentino et al. supplementary material [file S1478951525101156sup002.jpg]
